# Joint level-set and spatio-temporal motion detection for cell segmentation

**DOI:** 10.1186/s12920-016-0206-5

**Published:** 2016-08-10

**Authors:** Fatima Boukari, Sokratis Makrogiannis

**Affiliations:** Department of Physics and Engineering, Delaware State Univ., 1200 N. DuPont Hwy, Dover, 19901 DE USA

**Keywords:** Cell segmentation, Level sets, Nonlinear diffusion, Density estimation

## Abstract

**Background:**

Cell segmentation is a critical step for quantification and monitoring of cell cycle progression, cell migration, and growth control to investigate cellular immune response, embryonic development, tumorigenesis, and drug effects on live cells in time-lapse microscopy images.

**Methods:**

In this study, we propose a joint spatio-temporal diffusion and region-based level-set optimization approach for moving cell segmentation. Moving regions are initially detected in each set of three consecutive sequence images by numerically solving a system of coupled spatio-temporal partial differential equations. In order to standardize intensities of each frame, we apply a histogram transformation approach to match the pixel intensities of each processed frame with an intensity distribution model learned from all frames of the sequence during the training stage. After the spatio-temporal diffusion stage is completed, we compute the edge map by nonparametric density estimation using Parzen kernels. This process is followed by watershed-based segmentation and moving cell detection. We use this result as an initial level-set function to evolve the cell boundaries, refine the delineation, and optimize the final segmentation result.

**Results:**

We applied this method to several datasets of fluorescence microscopy images with varying levels of difficulty with respect to cell density, resolution, contrast, and signal-to-noise ratio. We compared the results with those produced by Chan and Vese segmentation, a temporally linked level-set technique, and nonlinear diffusion-based segmentation. We validated all segmentation techniques against reference masks provided by the international Cell Tracking Challenge consortium. The proposed approach delineated cells with an average Dice similarity coefficient of 89 % over a variety of simulated and real fluorescent image sequences. It yielded average improvements of 11 % in segmentation accuracy compared to both strictly spatial and temporally linked Chan-Vese techniques, and 4 % compared to the nonlinear spatio-temporal diffusion method.

**Conclusions:**

Despite the wide variation in cell shape, density, mitotic events, and image quality among the datasets, our proposed method produced promising segmentation results. These results indicate the efficiency and robustness of this method especially for mitotic events and low SNR imaging, enabling the application of subsequent quantification tasks.

## Background

Cell identification, quantification and characterization using imaging techniques are emerging research areas that are systematically integrated in biological and medical studies [1]. Recent developments in time-lapse microscopy enable the observation and quantification of cell-cycle progression, cell migration, and growth control [2]. The tasks of detecting and tracking individual cells or particles in a time series of images are key elements in this process. More importantly, the large volume of data produced by fluorescence microscopy and imaging modalities emphasizes the need for automated and robust techniques that can address the challenges in accurate detection and segmentation as well as tracking.

Cell tracking methodologies involve the tasks of preprocessing, cell segmentation and motion tracking [3–9]. In this context, segmentation of cells is a particularly challenging task that has a direct impact on the overall quantification process. Image segmentation is a popular field in the domain of image analysis. More specifically, parametric [10] and nonparametric active contour models [11–14] have been widely used in development of bio-imaging and biomedical image analysis techniques. An interesting aspect in cell analysis methods is the relation between image quality and segmentation accuracy. Many segmentation methods address certain types of datasets; however, for low-quality images and different cell types and shapes, the same methods may yield varying levels of performance.

Earlier published works propose to use partial differential equation (PDE) models for heat diffusion to detect motion with applications to moving edge detection [15], and human assistive technologies [16]. Building upon previous ideas for estimating motion activity using spatio-temporal diffusion [16], in this work we develop and utilize a heat flow analogy model in the joint spatio-temporal domain and combine this process with a region-based level-set optimization approach for cell segmentation of images obtained by fluorescence microscopy. Spatial and temporal motion parameters of our model are estimated for each dataset and an optimal Parzen bandwidth parameter is experimentally determined for density estimation of edges and outliers in each dataset. High activity regions are initially detected by solving numerically a system of coupled spatio-temporal nonlinear partial differential diffusion equations on three consecutive frames. In order to obtain more stability in parameter choice, we apply a histogram transformation approach to match the reference background threshold from the intensity distribution of each three consecutive frames to an intensity distribution model learned from all frames of the sequence during the training stage. After this step, each video sequence frame is scaled and transformed into a sequence with background with same order of magnitude for a more robust and a less sensitive parameter choice method. Then spatial and temporal motion parameters of our model are estimated for each dataset and an optimal Parzen bandwidth parameter is experimentally estimated for density estimation for edges and outliers for each dataset. After the spatio-temporal diffusion stage is completed, we compute the edge map by nonparametric density estimation using Parzen kernels. This process is followed by watershed-based segmentation to detect the moving cells. Next, adjacent regions with motion are merged to form a moving cell by mean intensity thresholding of these regions. Thresholding determines the boundary of each cell. Finally, the moving delineation curve is used as an initial level-set to be refined using a region-based process for final segmentation. We validated the joint approach denoted by ST-Diff-TCV over a set of sequences against reference data and compared the segmentation accuracy of the joint spatio-temporal and level-set technique with results derived from Chan-Vese (CV) segmentation [17], a temporally linked level set method that we have recently presented [18] denoted by TCV, and spatio-temporal diffusion based segmentation only (ST-Diff). Our method can accurately detect fluorescent cells at an average Dice coefficient rate of 89 % showing a clear improvement over region-based level set segmentation with and without temporal linking, and nonlinear diffusion-based segmentation. In addition, it can detect and segment newly appearing cells. Another feature of this method is it can detect cells hardly detectable by means of mean intensity and produces accurate results for high or low cell density images. This method allows to detect cells that were impossible to detect using the region based CV segmentation because the optimization criterion is defined by the mean intensity inside and outside the level set defined moving curve. Hence, cell regions with low intensity values are considered as part of the background, and the region competition process fails to delineate these cells. However, these regions are detected by the spatio-temporal motion detection method because they are rather detected by their high activity process than by their intensity value, then refined by CV model to detect the cell boundaries more accurately.

The structure of this paper is organized as follows: In Section ‘Methods’ we introduce the frame intensity standardization approach by histogram transformation, the spatio-temporal diffusion-based technique, followed by the detection of spatio-temporal discontinuities by Parzen density estimation, cell delineation and identification from background, followed by the region-based level-set model with temporal linking, and finally the joint spatio-temporal diffusion and temporal level-set region-based method. Section ‘Results and discussion’ describes the dataset properties, image quality assessment, performance validation with comparisons between CV, TCV, ST-Diff, and ST-Diff-TCV, followed by discussion of results. Finally, in the conclusion we summarize the main observations about the advantages of this method and perspectives for future work.

## Methods

### Frame intensity standardization by histogram transformation

PDE-based techniques calculate differential approximations; therefore they are sensitive to variations in pixel intensity ranges. The main objective of this stage is first to reduce intensity variations between frames of each sequence, and second, to obtain a robust intensity prior for the cell delineation process. We apply a histogram transformation approach to match the intensity distribution of each three consecutive frames defined by (1) to an intensity distribution model learned from all frames of the sequence during the training stage.

1 $$ P_{3F}(I) = {\lim}_{N_{total} \to \infty} \frac{N(I)}{N_{total}}, \quad F_{3F}(k) = {\int_{0}^{k}} P_{3F}(I) dI  $$

**Fig. 1:**
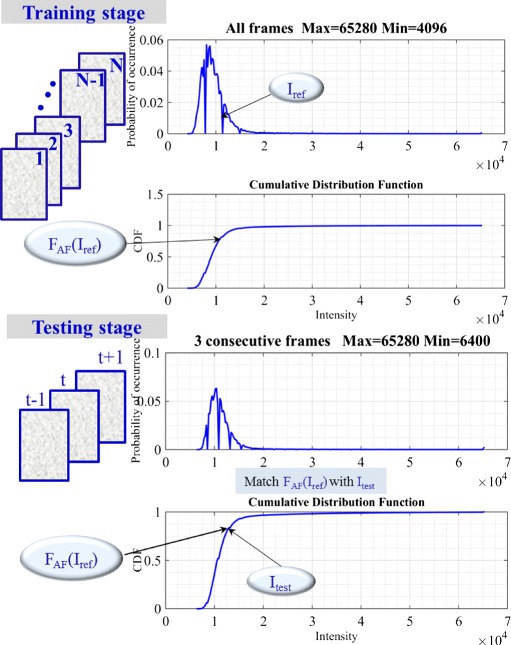
(Training stage) Probability density function of 48 frames of C2DL −MSC02 dataset and cumulative distribution function. (Testing stage) Normalized PDF and CDF of three consecutive frames of C2DL −MSC02 dataset

The general idea is to transform the frame intensities so that the reference cell/background threshold *I*_*ref*_ determined from the *F*_*AF*_ as expressed in (2) matches the global CDF reference *F*_*AF*_(*I*_*ref*_) corresponding to the *I*_*ref*_ value (2) indicating the tail of background intensity distribution of the complete sequence as displayed in Fig. 1 (top).

2 $$ P_{AF}(I) = {\lim}_{N_{total} \to \infty} \frac{N(I)}{N_{total}}, \quad F_{AF}(k) = {\int_{0}^{k}} P_{AF}(I) dI  $$

We aim to find a transformation so that the output image is a similar image that has a background value with the same order of brightness of the input image. Figure 1 (bottom) displays how we can determine the *I*_*test*_ value from the PDF of each three consecutive frames at the training stage using the prior value *F*_*AF*_(*I*_*ref*_) as expressed by (3).

3 $$ I_{test} = arg \min_{i} \vert F_{3F}(I(\omega)) - F_{AF}(I_{ref}) \vert,  $$

where *ω*∈*Ω*_3*F*_, $I : \mathbb {Z}^{2} \rightarrow \mathbb {R}^{+}$. Using these values, the resulting images are scaled and defined over the [ 0−255] range and with respect to the global minimum and global maximum intensities of all the frames of the dataset sequence after applying Eqs. 4, (5) and (6).

4 $$ T_{1}(I) = \frac{I_{ref} - G_{Min}}{I_{test} - L_{Min}} (I - G_{Min}) + G_{Min}  $$

5 $$  T_{2}(I) = \frac{255 \cdot (I - G_{Min})}{G_{Max} - G_{Min}}  $$

6 $$ I_{S}(\omega) = T(I(\omega)) = (T_{2} \circ T_{1}) (I(\omega)), \quad \omega \in \Omega_{3F}  $$

We experimentally found that the matched frames are less sensitive to the temporal, spatial diffusion parameters and Parzen kernel bandwidth values than the raw frames. In our experiments we used 256 bins for all datasets.

The following steps define the two algorithms that learn the CDF reference value for background intensity *F*_*AF*_(*I*_*ref*_) at the training stage (Algorithm 1) and transform every source image at the testing stage (Algorithm 2) so as to make its testing background as close as possible to the reference intensity.





**Fig. 2:**
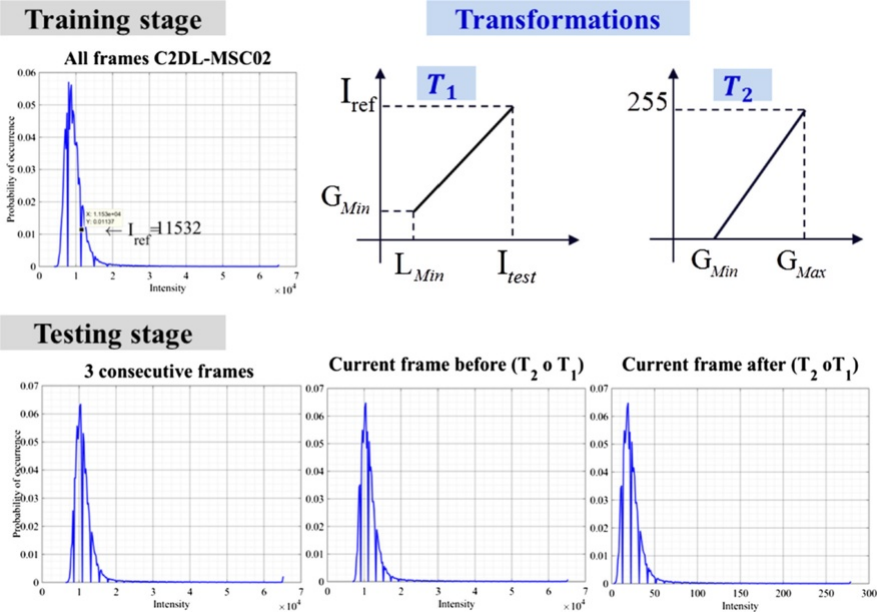
Histogram of all concatenated images of the C2DL-MSC02 dataset, and linear transformations and scaling of each pixel of the image (*top row, left to right*). The histogram of three consecutive frames that will be matched to the training dataset, and the histograms of the current frame before and after the transformation *T*(*I*(*ω*))=(*T*_2_∘*T*_1_)(*I*(*ω*)), *ω*∈*Ω*_3*F*_ (*bottom row, left to right*)

Figure 2 displays an intensity standardization example applied on three consecutive frames. The top row displays the histogram of the complete dataset and transformations *T*_1_ and *T*_2_ given by (4) and (5) respectively. The bottom row shows the histogram of 3 frames used to determine *I*_*test*_, the original histogram of currently processed frame and transformed histogram after applying (6).

We note that after the histogram transformation and scaling, all frames of the same sequence are going to have similar pixel intensity ranges.

### Spatio-temporal diffusion

#### Perona-Malik anisotropic diffusion

Diffusion algorithms perform image restoration by finding numerical solutions of the heat diffusion PDE [19, 20]. In this framework, the linear diffusion model is equivalent to applying Gaussian filtering to the image. To avoid the blurring and localization problems of linear diffusion filtering, Perona and Malik [21] proposed to replace the classic isotropic diffusion equation with the nonlinear diffusion model, which is based on the following PDE:

7 $$  \frac{\partial I }{\partial s} = div \left[ g \left({\vert \nabla I(x,y,s)} \vert \right) \cdot \nabla I(x,y,s) \right]  $$

where *I* is the image intensity, *s* the scale variable for 2D case, and *g*(·) a function that determines the amount of diffusion, also known as diffusivity function. This function is chosen to satisfy ${\lim }_{x \to \infty } g(x) \to 0$ so that diffusion is attenuated across edges. This function controls the amount of diffusion according to the edgestrength. Common options for *g*(·) are the sigmoid and exponential functions also reported by Perona and Malik in [21]:

8 $$  g(x) = \frac{1}{\left(1 + \frac{x^{2}}{k^{2}} \right)}  $$

9 $$  g(x) = e^{\left(- \frac{x^{2}}{k^{2}} \right)}  $$

where *k* denotes the conductance parameter that is a positive constant. The anisotropic diffusion method has been extensively used for image restoration as it largely preserves edge image features.

Moving regions are initially detected in each three consecutive frames by numerically solving the spatio-temporal partial-differential diffusion equation [18] where the diffusivity function is applied to the gradient magnitude of the image *I*. In this work we used the function (9) that is more suitable for region oriented applications [22]. This nonlinear diffusion is bound to the gradient magnitude [23]. It applies more diffusion in uniform regions and slows down at edges, therefore preserves high contrast edges over low contrast ones.

#### Spatio-temporal nonlinear diffusion

##### Partial differential equation model

Here we propose to simulate nonlinear heat flow through the processed frames in both spatial and temporal dimensions. This operation smooths-out the background regions and simultaneously preserves the spatio-temporal discontinuities corresponding to cells. More specifically, given 3 consecutive frames of the sequence at times {*t*−1,*t*,*t*+1}, we define a system of three coupled PDEs for each frame.

*At time points τ*={*t*−1,*t*,*t*+1}

10 $$ \begin{aligned}  \frac{\partial I(i,j,\tau,s)}{\partial s} = & g(\vert \nabla I(i,j,\tau,s) \vert) \cdot \Delta I(i,j,\tau,s) \\ & + \nabla g(\vert \nabla I(i,j,\tau,s) \vert) \cdot \nabla I(i,j,\tau,s) \end{aligned}  $$

*Initial condition*

11 $$ I(i,j,\tau,0) = I_{0}(i,j,\tau)  $$

*Boundary condition*

12 $$ \frac{\partial I}{ \partial \vec{n}} = 0 \qquad \text{on} \partial \Omega \times \partial T \times (0, S).  $$

##### Numerical solution

We used the Finite Difference method to solve the system of (10) on a 2D square grid lattice. We applied padding by replicating the pixel intensities at the image borders to obtain a zero gradient at the boundaries. This will enable the detection and localization of motion within each 3 consecutive frames.

*At t*

13 $$ {}\begin{aligned} I_{i,j,t}^{s+1} = I_{i,j,t}^{s} + \lambda_{s} &\left[ g\left(\vert \nabla I_{i+1,j,t}^{s} \vert\right) \cdot N_{t} + g\left(\vert \nabla I_{i-1,j,t}^{s} \vert\right) \cdot S_{t}\right.\\ & +\left. g\left(\vert \nabla I_{i,j+1,t}^{s} \vert\right) \cdot E_{t} + g\left(\vert \nabla I_{i,j-1,t}^{s} \vert\right) \cdot W_{t}\right]\\ & + \lambda_{t} \left[\nabla I_{i,j,t-1}^{s} \cdot PF + \nabla I_{i,j,t+1}^{s} \cdot NF\right] \end{aligned}  $$

*Att*−1

14 $$ \begin{aligned} I_{i,j,t-1}^{s+1} = I_{i,j,t-1}^{s} + \lambda_{s} &\left[g \left(\vert \nabla I_{i+1,j,t-1}^{s} \vert\right) \cdot N_{t-1}\right.\\ & + g\left(\vert \nabla I_{i-1,j,t-1}^{s} \vert\right) \cdot S_{t-1}\\ & + g\left(\vert \nabla I_{i,j+1,t-1}^{s} \vert\right) \cdot E_{t-1} \\ & + \left.g\left(\vert \nabla I_{i,j-1,t-1}^{s} \vert\right) \cdot W_{t-1}\right]\\ & - 2 \lambda_{tPF} \cdot \nabla I_{i,j,t-1}^{s} \cdot PF \end{aligned}  $$

*Att*+1

15 $$ \begin{aligned} I_{i,j,t+1}^{s+1} = I_{i,j,t+1}^{s} + \lambda_{s} & \left[g\left(\vert \nabla I_{i+1,j,t+1}^{s} \vert\right) \cdot N_{t+1}\right. \\ & + g\left(\vert \nabla I_{i-1,j,t+1}^{s} \vert\right) \cdot S_{t+1}\\ & + g\left(\vert \nabla I_{i,j+1,t+1}^{s} \vert\right) \cdot E_{t+1}\\ & + \left. g\left(\vert \nabla I_{i,j-1,t+1}^{s} \vert\right) \cdot W_{t+1}\right]\\ & - 2 \lambda_{tNF} \cdot \nabla I_{i,j,t+1}^{s} \cdot NF \end{aligned}  $$

where

16 $$ N_{t} = I_{i-1,j,t}^{s} - I_{i,j,t}^{s}, \quad S_{t} = I_{i+1,j,t}^{s} - I_{i,j,t}^{s}  $$

17 $$ W_{t} = I_{i,j-1,t}^{s} - I_{i,j,t}^{s}, \quad E_{t} = I_{i,j+1,t}^{s} - I_{i,j,t}^{s}  $$

18 $$ PF = I_{i,j,t-1}^{s} - I_{i,j,t}^{s}, \quad NF = I_{i,j,t+1}^{s} - I_{i,j,t}^{s}  $$

In (13), (14), and (15) *λ*_*s*_, *λ*_*t*_, *λ*_*tPF*_, *λ*_*tNF*_ denote the numerical “time” steps for spatial, temporal, next frame temporal, and previous frame temporal terms respectively. In our implementation we set *λ*_*t*_=*TS*_*Ratio*_·*λ*_*s*_ and *λ*_*tPF*_=*λ*_*tNF*_, where *TS*_*Ratio*_ is a fixed parameter for the ratio of temporal to spatial diffusion. The diffusivity function is applied to the gradient magnitude of the image *I*.

### Detection of spatio-temporal discontinuities by Parzen density estimation

The idea is to estimate the likelihood of mean intensity in the neighborhood of each pixel in the diffused frame. Assuming a model of unimodal probability density function (PDF) for region interiors and bimodal PDF for edges, we use the likelihood of mean intensity as an index of edge occurrence. Low values of this index correspond to a bimodal PDF indicating an edge. We estimate this likelihood by the nonparametric technique of Parzen kernels [24–26].

**Fig. 3:**
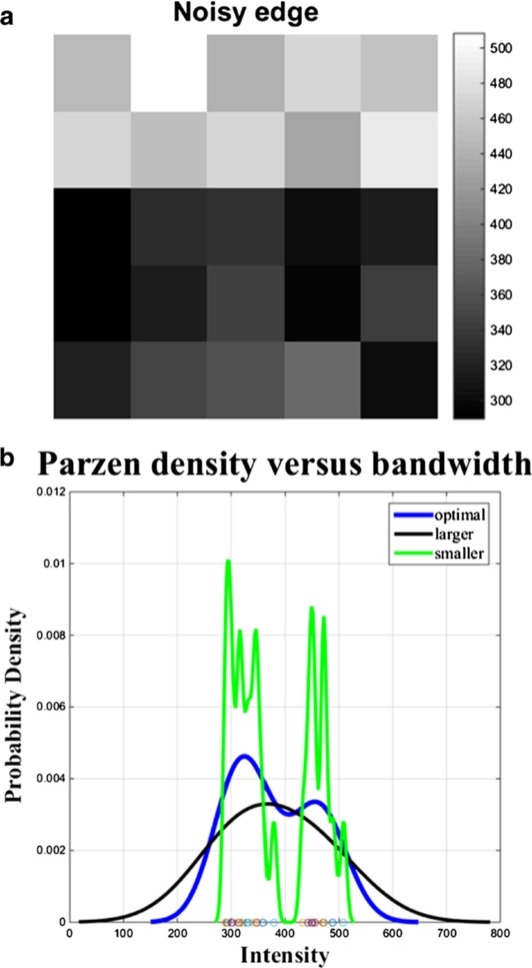
An example of **a** noisy edge detection using **b** nonparametric density estimation. Comparison of the Parzen density estimate for different bandwidth values of *h* on the same image intensity samples plotted on the horizontal axis. The optimal *h* value estimates the bimodality of the local intensity distribution. Use of smaller *h* is susceptible to statistical variability, while larger *h* will reduce the estimation accuracy

The Parzen density estimation belongs to the nonparametric density methods [23] i.e. methods to estimate the probability density function of a random variable that do not impose any initial assumptions about the shape of the probability density functions. Its operation is based on placing at each observation sample a probability mass and producing a potential according to a Gaussian kernel. The contributions of all the sample points are averaged to estimate the density value at every point of the image [25].

19 $$  f_{h} (x) = 1/(n \cdot h^{p}) \sum\limits_{i=1}^{n} K((x-x_{i})/h)  $$

where (*x*1,*x*2,,*xn*) is an independent and identically distributed sample drawn from some distribution with an unknown density *P*, *K*(·) is the kernel and *h*>0 is a smoothing parameter called the bandwidth. We can see in (19) that the kernel-bandwidth *h* can strongly affect the PDF estimate, especially when the number of observations *n* is finite. Very small *h* values will produce a ragged density estimate, while very large values will smooth the structure of the PDF. An optimal *h* value is usually experimentally determined to find a compromise between the variability and accuracy and converge towards the true PDF. Figure 3 shows three density estimates: the green solid line corresponds to a small bandwidth, the black line corresponds to a large bandwidth, while the blue line represents a bandwidth selection that produces a more accurate estimate of the underlying bimodal distribution.

### Cell delineation and identification

The edge map can be interpreted as a topographic surface consisting of valleys corresponding to spatio-temporally homogeneous areas and peaks denoting spatio-temporal discontinuities. The next step is to apply watershed segmentation. Watershed analysis has emerged from mathematical morphology and was implemented by a series of morphology operators in its early versions. Since then, several implementations have appeared, proposing iterative, sequential, arrowing, flow line oriented and flooding techniques [27]. Regional minima of the topographic relief are selected and flooded to form the moving regions. We obtain a watershed region in the resulting segmentation for each minimum. We first find the watershed ridges of the stochastic map of spatio-temporal discontinuities. The watershed transform divides a multivalued image into separate regions by identifying the regional minima and applying flooding operations to each minimum to fill the watershed basins. Each basin corresponds to a region. We first invert the stochastic map produced by Parzen density estimation to form regions separated by spatio-temporal discontinuities.

To separate the cells we calculate intensities and areas of watershed regions and classify them into cells or background using area and intensity prior information and likelihoods *p*(*area*|*c*_*i*_), *p*(*I*|*c*_*i*_) in Gaussian form, where *c*_*i*_={*background*,*cell*}. Adjacent watershed regions with coherent motion should be merged together to form a moving object. We compute mean intensity over the watershed regions and classify into foreground or background using as threshold value the standardized reference value *T*(*I*_*ref*_) calculated by (6).

### Region-based level-set model with temporal linking

In contrast to edge based methods like classical snakes [10] and early level-set methods [11], where an edge detector is used to stop the evolving curve, region-based methods tend to be less sensitive to noise. The use of region-based statistics may prove advantageous for images characterized by edge discontinuity and higher level of noise. Chan-Vese (CV) method [17] is a region-based active contour model for energy minimization. Here, we shortly describe the theoretical background of Chan-Vese model and its minimization framework. This model is a special case of the Mumford-Shah functional [28] for segmentation using piecewise constant approximation.

This model segments an input scalar image *I*(*x*,*y*) with $I:\Omega \to \mathbb {R}$ and $(x,y) \in \Omega \subset \mathbb {R}^{2}$ into two disconnected regions *Ω*_1_ and *Ω*_2_ representing the foreground and background respectively of low intra-region variance and separated by a smooth closed contour *C* such that *Ω*=*Ω*_1_∪*Ω*_2_∪*C*. Chan and Vese proposed to use level-set functions to solve this optimization problem. In the level-set method, the contour is represented as the zero level-set of a Lipschitz function $\phi : \Omega \to \mathbb {R}$, where *ϕ* is positive inside *C* and negative outside *C*. Segmentation is obtained by minimizing the following energy functional in terms of level-set:

20 $$ \begin{aligned} F(\phi,c_{1},c_{2}) = & \mu \cdot length\{\phi=0\} + v \cdot area \{ \phi \geq 0 \}\\ & + \lambda_{1} \int_{\phi \geq 0} {\vert I - c_{1} \vert}^{2} dx dy\\ & + \lambda_{2} \int_{\phi < 0} {\vert I - c_{2} \vert}^{2} dx dy \end{aligned}  $$

where *C* is the evolving curve, *c*_1_ and *c*_2_ are the average intensity levels inside and outside the contour *C*, and *μ*,*ν*,*λ*_1_,*λ*_2_≥0 are energy weights. The length and area of *C* are regularizing terms are formulated using the Heaviside *H* and Dirac *δ* functions. In [17] the Euler-Lagrange equations and the gradient-descent method were used to derive the following evolution equation for the level-set function *ϕ* that minimizes the fitting energy using time to parametrize the gradient descent:

21 $$\begin{array}{*{20}l}  {}\frac{\partial \phi (t,x,y)}{\partial t} = & \delta(\phi(x,y)) \cdot \left[ \mu \cdot div \left(\frac{\nabla \phi(x,y)}{\vert \nabla \phi(x,y) \vert} \right) - v \right.  \\ & -\left. \lambda_{1} {(I-c_{1})}^{2} + \lambda_{2} {(I-c_{2})}^{2}{\vphantom{\sum\limits{1}}} \right] \in (0, \infty) \times \Omega \end{array} $$

with initial and Neumann boundary conditions

22 $$ \phi(0,x,y) = \phi_{0}(x,y) \in \Omega  $$

23 $$ \frac{\delta(\phi)}{\vert \nabla \phi \vert} \cdot \frac{\partial \phi}{\partial \vec{n}} = 0 \in \partial \Omega  $$

#### Temporally linked level-set segmentation

This approach makes use of temporal connection between consecutive level-set results [17]. That is, when segmenting an image, which is a part of a temporal sequence, we make use of the level-set results reached from minimization of the global energy associated with the contours of the segmented cells found in the previous time point

24 $$ \phi_{n+1} (x,y;0) = \phi_{n} (x,y; i_{final}), \forall (x,y) \in \Omega,  $$

where *n* is the frame number in the time-lapse sequence, and *i*_*final*_ is the number of iterations required to converge for frame *n*. We take the contour result of each frame as the initial contour for the following one. These results are utilized to minimize the energy functional of the next image. If the segmentation in frame *n* is accurate, then this initialization will correspond to a point close to the global optimum of the energy functional in frame *n*+1. The main steps of this technique are summarized in Algorithm 3.



### Joint spatio-temporal diffusion and temporally linked level-set approach (ST-Diff-TCV)

We propose a joint method combining the S-T differential information with the high delineation accuracy that characterizes level set-based segmentation [15, 17]. More specifically, we use S-T Diffusion to delineate the cells first, then initialize TCV with the S-T Diffusion result to refine the cell segmentation. We apply the S-T Diffusion technique on each modulo *k* frame to address cell events that may not be handled by TCV such as cell mitosis, cell division, new cells entering the field of view, and other cases.

**Fig. 4:**
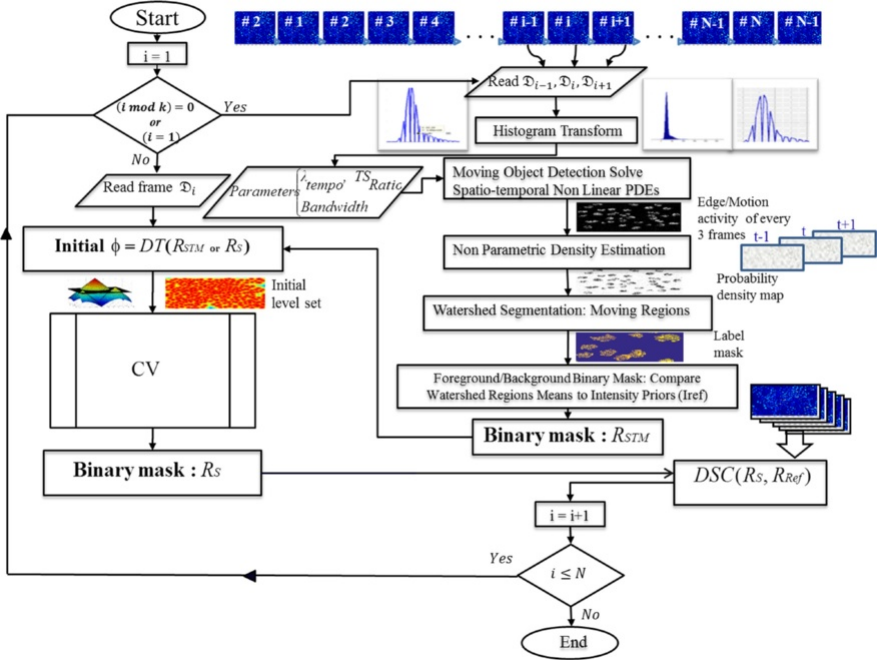
Outline of the proposed joint spatio-temporal nonlinear diffusion algorithm and temporally linked level sets methods (ST-Diff-TCV)

**Fig. 5:**
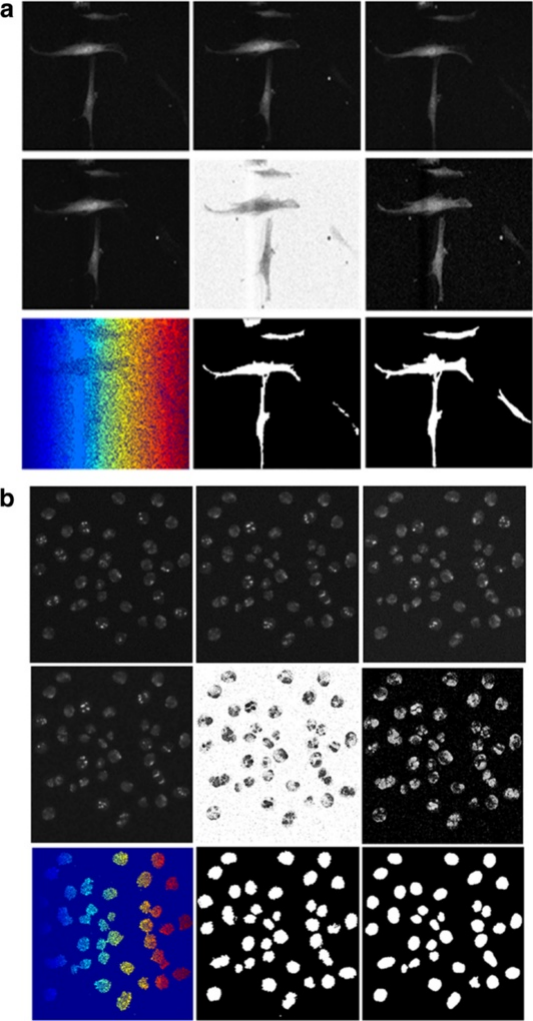
Intermediate results produced by ST-Diff-TCV on sample frames of **a** C2DL-MSC02 and **b** N2DH-SIM04 data sequences. *First row*: center, previous and next frames in the temporal space (left to right) *Second row*: S-T diffused frame, kernel density estimation of edge-moving regions then the inverted probability density map. *Third row*: watershed result, cell identification after foreground/background separation, and the reference segmentation mask (left to right)

This strategy may also reduce the computational cost by applying the S-T Diffusion technique to a limited number of frames. We apply these methods on several datasets of fluorescence microscopy images with varying levels of difficulty with respect to cell density, resolution, contrast, and signal-to-noise ratio. The flowchart in Fig. 4 outlines the main stages of our proposed technique. Furthermore, in Fig. 5 we display intermediate results from each stage on a test frame and its temporal neighbors for the C2DL-MSC02 and N2DH-SIM04 sequences.

## Results and discussion

### Data description

**Fig. 6:**
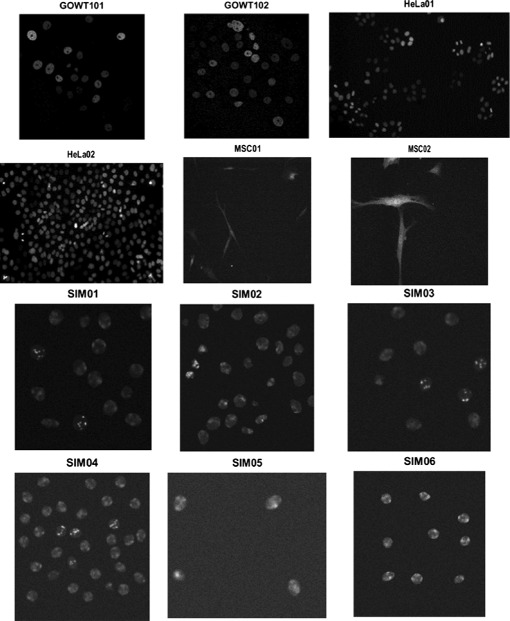
The 6 real and 6 simulated time-lapse sequences used for validation

The datasets consist of 2D fluorescent microscope time-lapse image sequences. We used 12 time-lapse video sequences; 6 real microscopy time-lapse sequences and 6 computer simulated videos with various cell densities and noise levels. We obtained the training and challenge data sets from the cell tracking challenge website [29]. Simulated videos: The 6 simulated videos displayed fluorescently labeled nuclei of the HL60 (human promyelocytic leukemia) cell line migrating on a flat 2D surface (N2DH-SIM01, N2DH-SIM02, N2DH-SIM03, N2DH-SIM04, N2DH-SIM05, N2DH-SIM06). They differ in the level of noise, cell density of the initial population, the number of cells leaving and entering the field of view and the number of simulated mitotic events, yielding up to 70 cells in the field of view [29]. Real videos: We used 3 datasets each containing 2 time-lapse sequences. Two video sequences named Fluo-C2DL-MSC01 and Fluo-C2DL-MSC02 with rat mesenchymal stem cells, 2 video sequences named N2DH-GOWT101 and N2DH-GOWT102 of mouse embryonic stem cells and N2DL-HeLa01 and N2DL-HeLa02 expressing HeLa cells. These datasets are considered to have high level of difficulty [29] because of the high cell density and low resolution and intensity. Summarized information on our test data is listed in Table 1, including the image matrix size, number of frames and level of difficulty. Furthermore, a sample frame of each dataset is displayed in Fig. 6.

**Table 1:** Image sequence properties and quality using Signal-to-Noise Ratio (SNR) and Contrast-to-Noise Ratio (CNR)

Dataset name	Average SNR std	Average CNR std	Level of difficulty
N2DH-SIM01	21.53 ± 0.69	7.96 ± 0.95	
N2DH-SIM02	22.21 ± 0.65	8.35 ± 0.96	*Medium:*different noise levels
N2DH-SIM03	18.59 ± 0.47	4.21 ± 0.47	cell density of the initial
N2DH-SIM04	18.97 ± 0.47	4.09 ± 0.49	population and number of
N2DH-SIM05	19.49 ± 0.54	4.22 ± 0.60	simulated mitotic events.
N2DH-SIM06	21.92 ± 0.54	7.90 ± 0.78	
C2DL-MSC01	14.67 ± 0.67	2.11 ± 0.36	*High:*low SNR, cell strectching appear
C2DL-MSC02	15.09 ± 2.31	4.47 ± 1.49	as discontinuous extensions of the cells.
N2DL-HeLa01	26.60 ± 3.41	19.23 ± 7.67	*High:*high cell density, low resolution,
N2DL-HeLa02	16.02 ± 1.67	5.40 ± 1.08	frequent mitoses (normal and abnormal).
N2DH-GOWT101	22.47 ± 0.49	12.62 ± 0.77	*Medium:*heterogeneous staining, prominent nuclei,
N2DH-GOWT102	18.91 ± 0.92	8.32 ± 0.91	mitoses, cells entering and leaving the field of view

### Image quality assessment of the datasets

In our first experiment, we measured the image quality of our datasets and then evaluated the segmentation accuracy. We utilized the available reference data for this purpose. The reference data consist of manually annotated videos for segmentation and tracking along with a short description and links to the raw datasets obtained from [29]. We first used the reference data to estimate the average Signal-to-Noise Ratio (*SNR*) and Contrast-to-Noise Ratio (*CNR*) of each dataset. The *SNR* and *CNR* measures are defined as follows:

25 $$  SNR = 20 \log_{10} \frac{\bar{u}_{C}}{\bar{u}_{B}}  $$

26 $$  CNR = \frac{\vert \bar{u}_{C} - \bar{u}_{B} \vert}{\sigma_{B}}  $$

where $\bar {u}_{C}$ is the average image intensity over the cell regions, $\bar {u}_{B}$ is the average intensity over the background and *σ*_*B*_ is the standard deviation of the background pixels. In Table 1 we list the average *SNR* (in dB) and average *CNR* that are means over all frames in each sequence using (25) and (26) and corresponding standard deviations of each dataset over cell regions. A comparison between the qualitative level of difficulty and the image quality metrics in Table 1 shows that the simulated sequences have higher *SNR* and *CNR*, therefore being more amenable to segmentation than the real sequences.

### Comparison of CV, TCV, ST-Diff, and ST-Diff-TCV methods

We applied the standard CV, TCV, ST-Diff, and ST-Diff-TCV methods on 12 time-lapse fluorescent microscopy datasets listed in Table 1. Fluorescent microscopy imaging is often times subjected to a mixture of different types of noise. The main goal of a preprocessing step is to reduce the corruption caused by noise and to improve the image quality [28]. To facilitate data analysis, a combination of filters and histogram enhancement is applied to the datasets to obtain better delineation accuracy.

We segmented each dataset using each method and evaluated the segmentation performance against reference masks. The main purpose is to evaluate how well the segmented cells match the cell regions of the reference mask. We quantify the accuracy of the segmentation performance by computing the DICE similarity coefficient denoted by *DSC*. This is defined as:

27 $$ DSC = 2 \times \frac{\vert R_{S} \cap R_{Ref} \vert}{\vert R_{S} \vert + \vert R_{Ref} \vert} \in\ [\!0,1],  $$

where *R*_*Ref*_ is the set of all pixels that belong to cell regions in the reference image, *R*_*S*_ is the set of all binary regions delineated by the tested segmentation technique. The DICE coefficient measures the relative similarity between two binary images over their cardinalities. It is frequently used for image segmentation validation. The value of 1 indicates perfect matching.

**Fig. 7:**
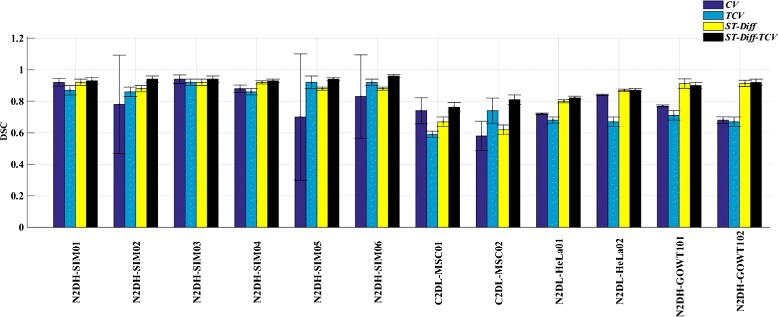
Dice similarity coefficients (*DSC*) produced by standard Chan −Vese model (CV), temporally linked Chan −Vese technique (TCV), spatio −temporal diffusion (ST −Diff), and the joint ST −*Diff*−TCV methods over all 12 datasets

We computed the DICE coefficient between the automated and reference segmentations for each method and for each dataset. Further, we computed the means and the standard deviations of the DICE similarity coefficients over all frames for each dataset sequence. Figure 7 and Table 2 report the *DSC* estimates and their variations for each sequence. In addition, the last row in Table 2 lists the overall *DSC* values for all datasets. In Fig. 7 and Table 2 we observe that ST-Diff-TCV yields higher *DSC* values for 11 out of the 12 test sequences. ST-Diff-TCV yields an average Dice coefficient of 0.89 over all datasets, while both CV and TCV yield 0.78, and ST-Diff yields 0.85 (Table 2). Furthermore, the standard deviation values in Table 2 show more robustness and stability. That is, the standard deviations obtained from ST-Diff and ST-Diff-TCV (0.01-0.03) are significantly smaller than those derived from the CV method (0.01-0.4) and even TCV (0.02-0.08) indicating better convergence and stability.

**Table 2:** The mean DICE coefficient obtained from segmentation of each sequence by CV, TCV, ST-Diff, and the joint ST-Diff-TCV method

Dataset name	Size	Frames	CV	TCV	ST-Diff	ST-Diff-TCV
N2DH-SIM01	494x534	56	0.92 ± 0.02	0.87 ± 0.03	0.92 ± 0.02	**0.93 ± 0.02**
N2DH-SIM02	569x593	100	0.78 ± 0.31	0.86 ± 0.03	0.88 ± 0.02	**0.94 ± 0.02**
N2DH-SIM03	606x605	100	**0.94 ± 0.03**	0.92 ± 0.02	0.92 ± 0.02	**0.94 ± 0.02**
N2DH-SIM04	673x743	56	0.88 ± 0.02	0.86 ± 0.02	0.91 ± 0.01	**0.93 ± 0.01**
N2DH-SIM05	597x525	76	0.70 ± 0.40	0.92 ± 0.04	0.88 ± 0.01	**0.94 ± 0.01**
N2DH-SIM06	655x735	76	0.83 ± 0.27	0.92 ± 0.02	0.88 ± 0.01	**0.96 ± 0.01**
C2DL-MSC01	992x832	48	0.74 ± 0.08	0.59 ± 0.02	0.67 ± 0.03	**0.76 ± 0.03**
C2DL-MSC02	1200x782	48	0.58 ± 0.09	0.74 ± 0.08	0.62 ± 0.03	**0.81 ± 0.03**
N2DL-HeLa01	1100x700	92	0.72 ± 0.01	0.68 ± 0.02	0.80 ± 0.01	**0.82 ± 0.01**
N2DL-HeLa02	1100x700	92	0.84 ± 0.01	0.67 ± 0.03	**0.87 ± 0.01**	**0.87 ± 0.01**
N2DH-GOWT101	1024x1024	92	0.77 ± 0.01	0.71 ± 0.03	**0.91 ± 0.03**	0.90 ± 0.02
N2DH-GOWT102	1024x1024	92	0.68 ± 0.02	0.67 ± 0.03	0.91 ± 0.02	**0.92 ± 0.02**
Mean *DSC*			0.78	0.78	0.85	**0.89**

Boldface denotes the top performing algorithm

**Fig. 8:**
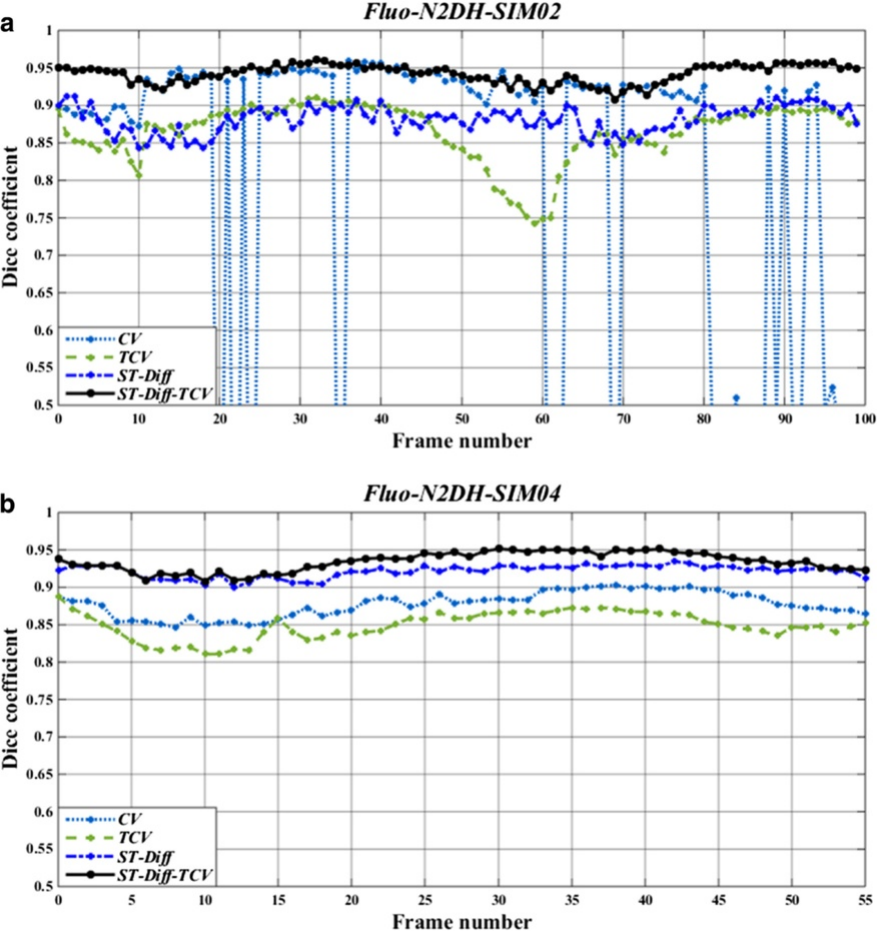
Dice similarity coefficients (*DSC*) produced by standard Chan-Vese segmentation (CV), temporally linked Chan-Vese technique (TCV), spatio-temporal diffusion (ST-Diff), and the joint ST-Diff-TCV methods for each frame of **a** N2DH-SIM02 and **b** N2DH-SIM04 datasets

To illustrate the performance comparison among the three tested methods in more detail, we show in Fig. 8 the results derived from CV, TCV, ST-Diff, and ST-Diff-TCV methods on N2DH-SIM02 and N2DH-SIM04 datasets. In the N2DH-SIM02 sequence (Fig. 8(a)) we observe that because of the non-convexity of the energy functional (allowing therefore many local minima), the CV method reached several local minima of energy. In contrast, the TCV method led to a global minimum of the energy. ST-Diff-TCV method yields accurate delineation of the cells with fewer fluctuations in the Dice coefficient than the other methods. We note that ST-Diff-TCV yields an average Dice coefficient of 0.94, while CV yields 0.78, TCV yields 0.86, and ST-Diff yields 0.88. In the N2DH-SIM04 dataset as displayed in Fig. 8(b) we observe that ST-Diff-TCV produces the highest accuracy at a *DSC* value of 0.93, followed by ST-Diff, CV and TCV with Dice coefficients of 0.91, 0.88 and 0.86 respectively.

**Fig. 9:**
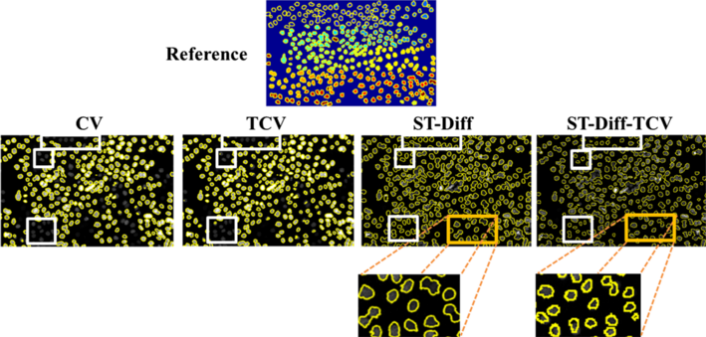
Cell boundaries produced by the 4 tested methods on N2DL-HeLa02 sequence frame. The spatio-temporal analysis enables the identification of more moving cells than the level-set models. Furthermore, ST-Diff-TCV produces more accurate cell separation than ST-Diff (magnified regions)

Furthermore, Fig. 9 displays cell delineations represented by yellow contour maps for one frame of the sequence N2DL-Hela2 including the manual reference, and automated segmentation produced by all tested methods. This sequence has an increased level of difficulty because of the high cell density and low contrast between some cells and the background. Because CV and TCV methods use piecewise constant approximations for object and background as can be seen in (21), the low contrast cells are likely to be falsely identified as background therefore reducing *DSC* (CV: 0.84, TCV: 0.67). On the other hand, both ST-Diff and ST-Diff-TCV identify the spatio-temporal discontinuities and detect the cells that are missed by CT and TCV as outlined by white rectangles in Fig. 9. In the magnified local regions of the test image we note that ST-Diff-TCV yields more accurate cell separation for adjacent cells than ST-Diff.

In some of our datasets, the fluorescent images contain low intensity nuclei where there is absorption rather than fluorescence in local parts of the same image resulting in heterogeneous cell intensity levels. These darker regions have low intensity that can be mis-detected as background. As a result the region competition process would fail to delineate them. However, these cells are identified by the spatio-temporal motion analysis method because of their high temporal activity, which is proven to be more efficient in these cases. For example, we observed that CV was not able to detect some cells with very low intensity in both N2DL-Hela and N2DH-GOWT sequences. Conversely, those cells were very well delineated using the temporal differences between frames, i.e., by ST-Diff-TCV, thus significantly improving the segmentation accuracy from 0.72 to 0.82 for N2DL-Hela01, and from 0.68 to 0.92 for N2DH-GOWT102 leading to *DSC* improvements up to 24 %.

On the other hand, the proposed technique involves some motion diffusion – i.e., *TS*_*Ratio*_, *λ*_*t*_, and Parzen kernel parameters – which are experimentally determined for each sequence. ST-Diff-TCV performance exhibits moderate sensitivity to the parameter values. In this work we performed exhaustive grid search in the parameter space to identify the optimal settings. Alternate parameter optimization techniques may be required to achieve more accurate segmentation in sequences with significantly different quality levels and cell types. In summary, our experiments suggest that the joint ST-Diff-TCV method improves the segmentation accuracy compared to CV, TCV, and ST-Diff, especially when applied to simulated and real microscopy images with cells characterized by wide intensity variations and undergoing mitotic events, changes in density, and low *SNR*.

## Conclusions

In this work, we introduced a local-global co-operative approach to dynamic cell segmentation. One component of this approach performs nonlinear spatio-temporal diffusion-based motion analysis, Parzen kernel-based detection of discontinuities, and watershed-based foreground-background separation. This local-based segmentation part generates a delineation that we use as the initial level-set in a region-based temporally linked level-set model. The improvement in segmentation accuracy is mainly achieved by using both the local motion and the global statistical information for segmenting cells with heterogeneous intensity levels. We evaluated the performance of our approach denoted by ST-Diff-TCV, two level-set based methods denoted by CV and TCV, and ST-Diff methods on datasets obtained from the online Cell Tracking challenge [29]. Every dataset addresses a different type of challenge for segmentation.

In comparison to CV and TCV, both ST-Diff and ST-Diff-TCV perform more robust cell segmentation, especially for cells undergoing mitosis, leaving and entering the field of view, and cells with lower mean intensity than the background intensity level. ST-Diff-TCV further improves the segmentation accuracy compared to ST-Diff by refining the cell delineation. Still, this method is dependent on some parameter retuning to optimize segmentation accuracy for different types of imaging sequences. This approach is beneficial for quantification of a wide range of types of image sequences. Future goals are to compute cell features representing cell morphology for classification and tracking.
